# Supplementary material for: Evolution and expansion of Li concentration gradient during charge–discharge cycling
Source: Nat Commun. 2021 Jun 21;12:3814. doi: 10.1038/s41467-021-24120-w (PMC8217543; doi:10.1038/s41467-021-24120-w)
Supplement: Supplementary file 1 — Supplementary Information [file 41467_2021_24120_MOESM1_ESM.pdf]

Supplementary Information

Evolution and expansion of Li concentration gradient during charge–discharge cycling

Byeong-Gyu Chae[1,$,*], Seong Yong Park[1,$,*], Jay Hyok Song[2], Eunha Lee[1], Woo Sung Jeon[1]

[1]*Analytical Engineering Group, Material Research Center, Samsung Advanced Institute of Technology, Samsung Electronics Co., Ltd., 130 Samsung-ro, Suwon 16678, Republic of Korea*

[2]*Energy1 Lab, Samsung SDI, Suwon 443-803, Republic of Korea*

[$]*These authors contributed equally: Byeong-Gyu Chae, Seong Yong Park.*

[*]*These authors jointly supervised this work: Byeong-Gyu Chae, Seong Yong Park.*

✉*e-mail: bg.chae@samsung.com; sydra.park@samsung.com*

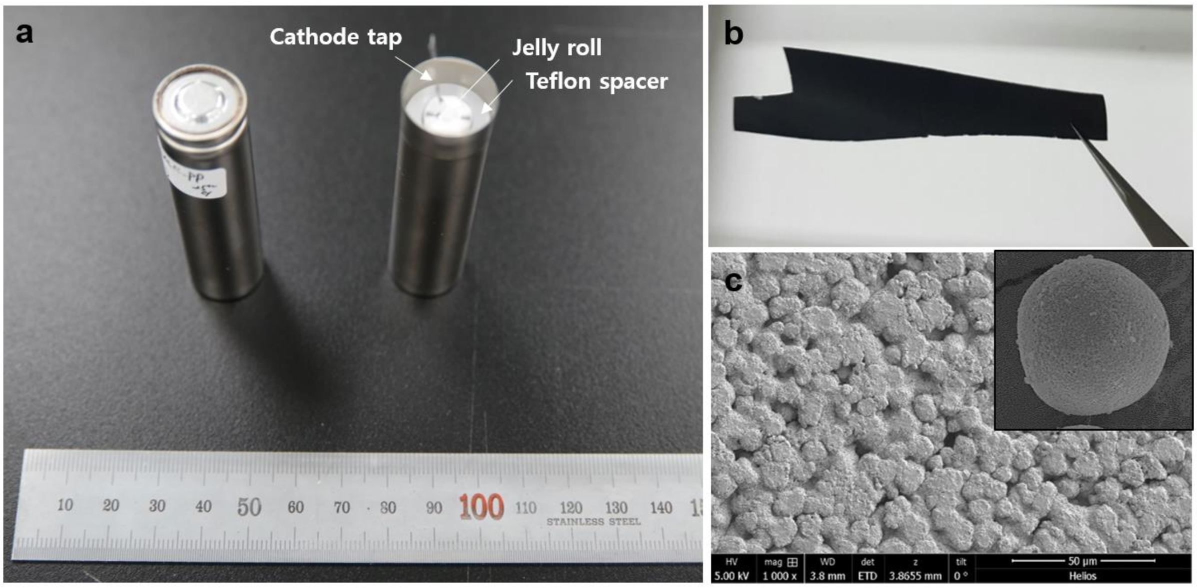

Supplementary Fig. 1. Photograph of 18650 mini full cells **(a)** and NCM cathode **(b).** SEM image of the surface of the NCM cathode **(c)**. Inset shows a spherical NCM secondary particle with a ~10 μm diameter.

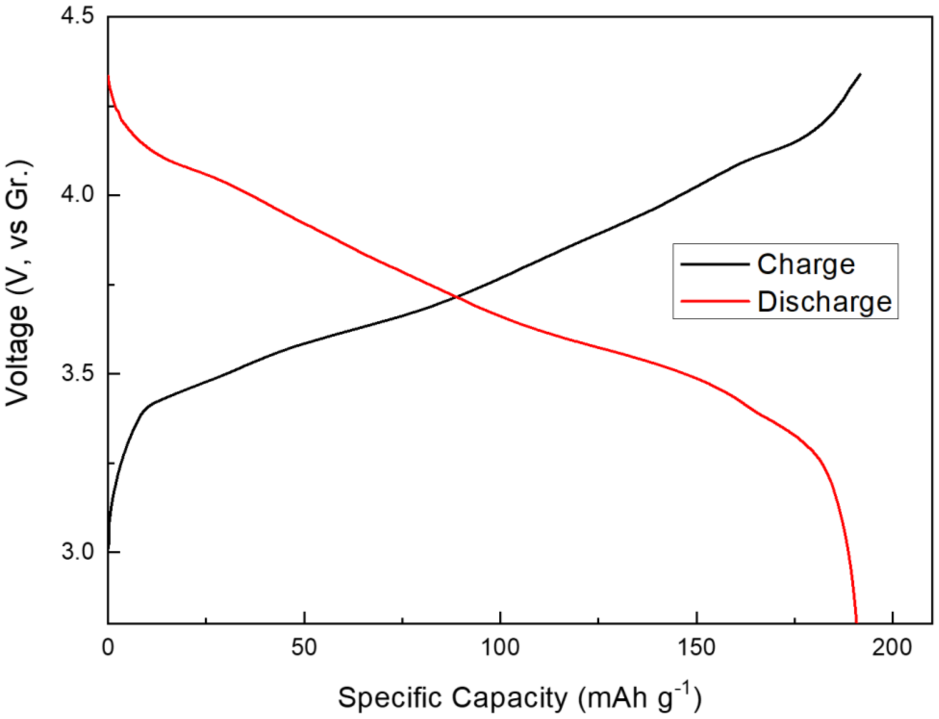

Supplementary Fig. 2. Electrochemical cycling performance of NCM. Charge and discharge curves of an NCM cell operated in the voltage range of 2.8–4.35 V at 0.2C and 45 °C.

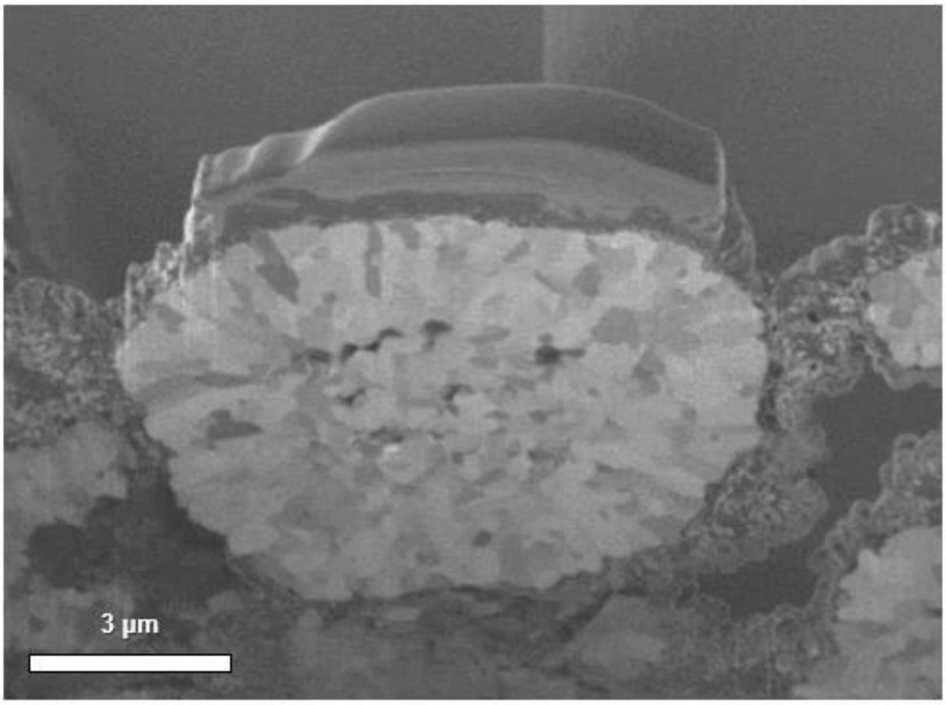

Supplementary Fig. 3. Cross-sectional scanning electron microscopy (SEM) images of an NCM primary particle.

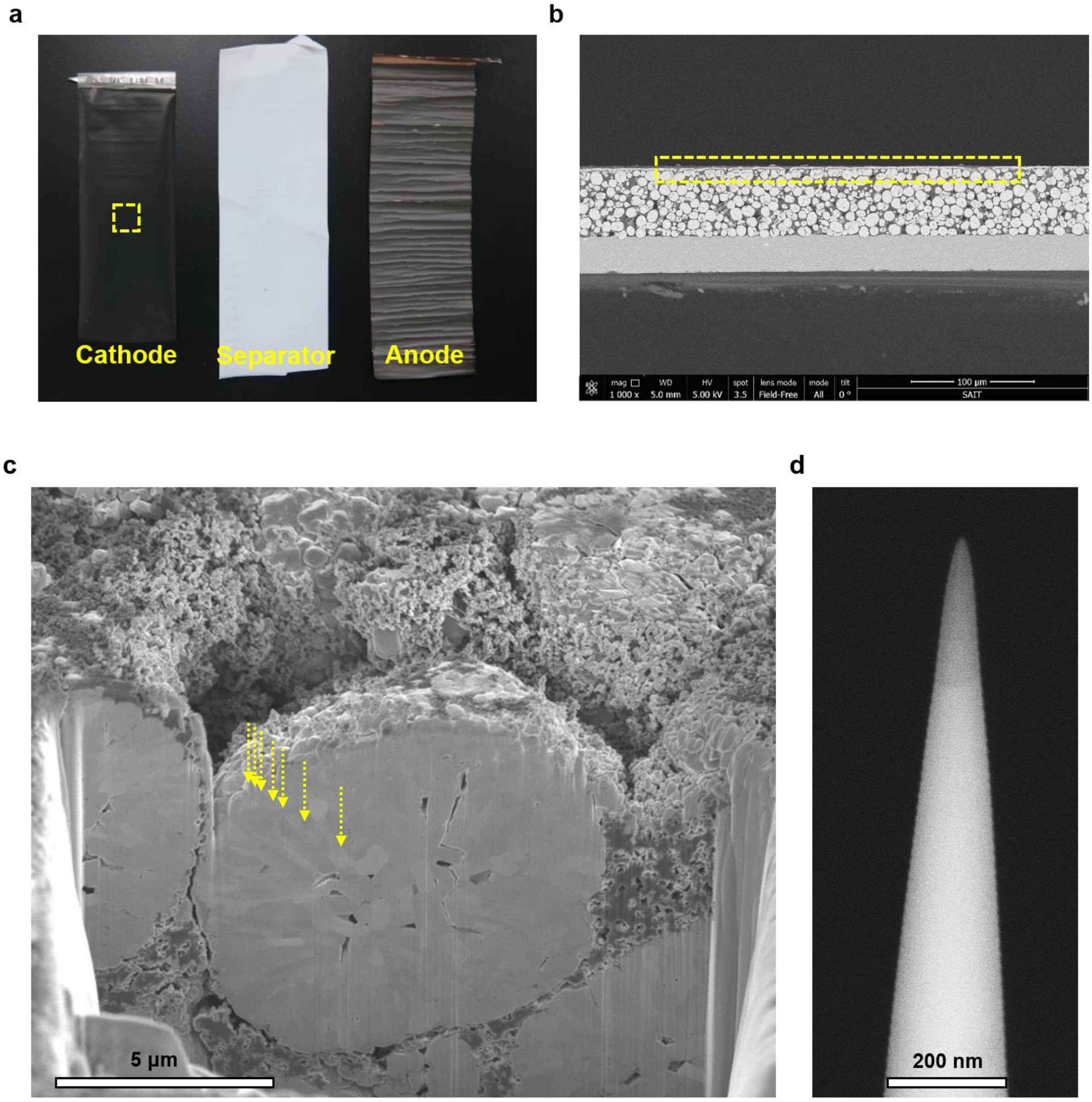

Supplementary Fig. 4. APT analysis regions in NCM. Image of a disassembled cell (a). Cross-sectional images of an NCM cathode fabricated by a cooling cross-section polisher (b) and an NCM particle sectioned by FIB (c). SEM image of the needle-shaped APT specimen after final milling (d). APT specimens were taken from the centre of the NCM cathode surface, as indicated by the yellow dotted rectangle. Then, the APT samples were fabricated as a function of the radial direction from the surface of the particle, as indicated by the yellow arrows. The distance from the surface of each sample was measured by FIB.

a

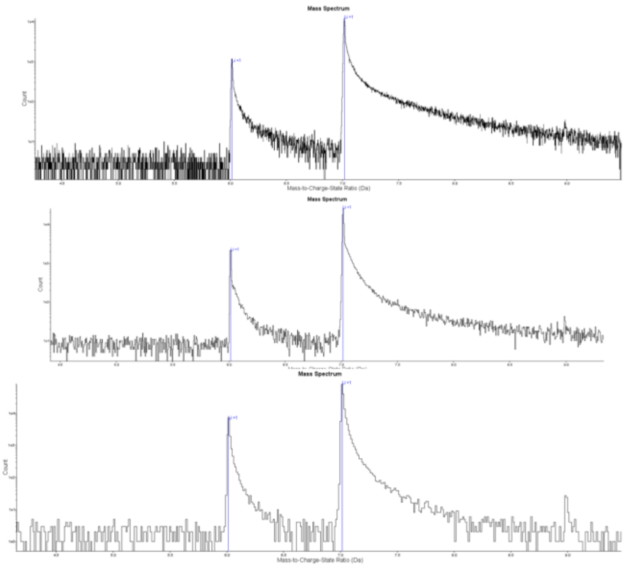

b

Specimen		Concentration
		Li
NCM-pristine		1.03 ± 0.016
NCM-formation		0.96 ± 0.012
NCM-100 cycles	R1 (<100 nm)	0.57 ± 0.013
	R2 (~200 nm)	0.68 ± 0.024
	R3 (~300 nm)	0.76 ± 0.011
	R4 (~2 μm)	0.99 ± 0.035
NCM-300 cycles	R0 (surface)	0.03 ± 0.004
	R1 (<30 nm)	0.37 ± 0.011
	R2 (~100 nm)	0.47 ± 0.016
	R3 (~350 nm)	0.69 ± 0.013
	R4 (~700 nm)	0.77 ± 0.027
	R5 (~1 μm)	0.84 ± 0.042
	R6 (~1.5 μm)	0.91 ± 0.001
	R7 (~2 μm)	1.03 ± 0.018

Supplementary Fig. 5. Isotopic ratio of Li (a). A negligible amount of Li deviates from the ideal isotopic ratio. Atomic fraction of Li (b) along the depth from the surface, as measured by APT.

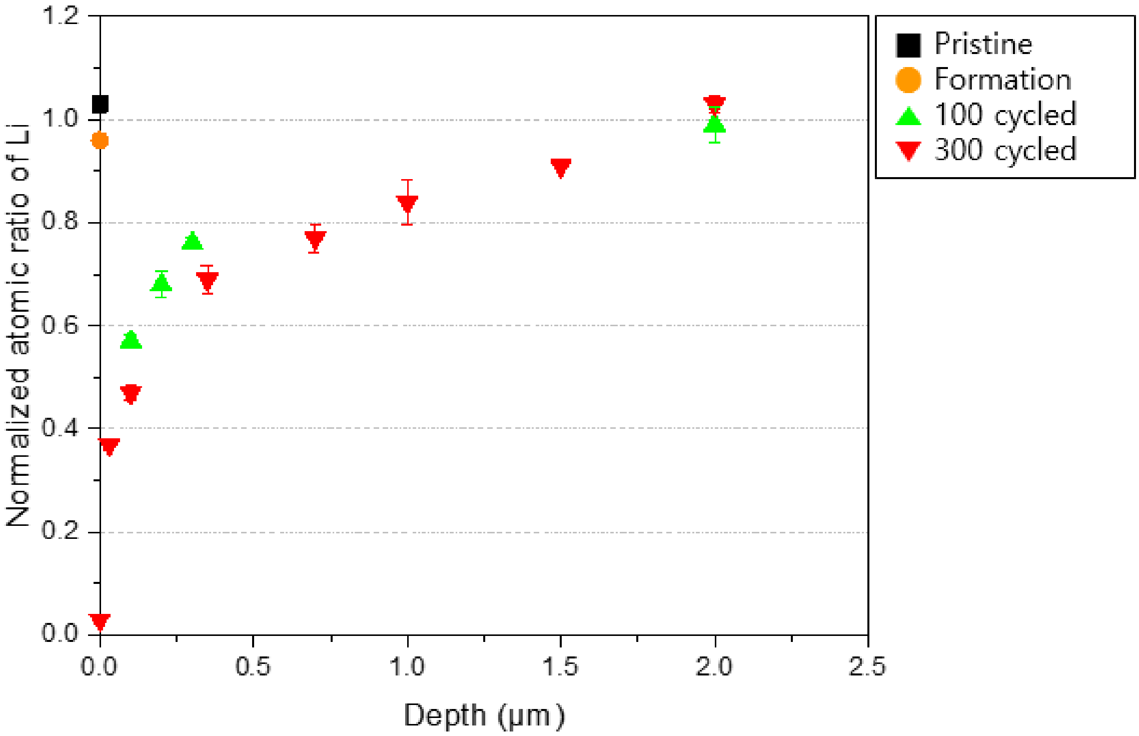

Supplementary Fig. 6. Normalised atomic ratio of Li measured by APT along the depth of the particles before and after 100 and 300 cycles. For direct comparison, the atomic ratio of each specimen was normalised and is expressed as a function of the approximate distance from the surface.

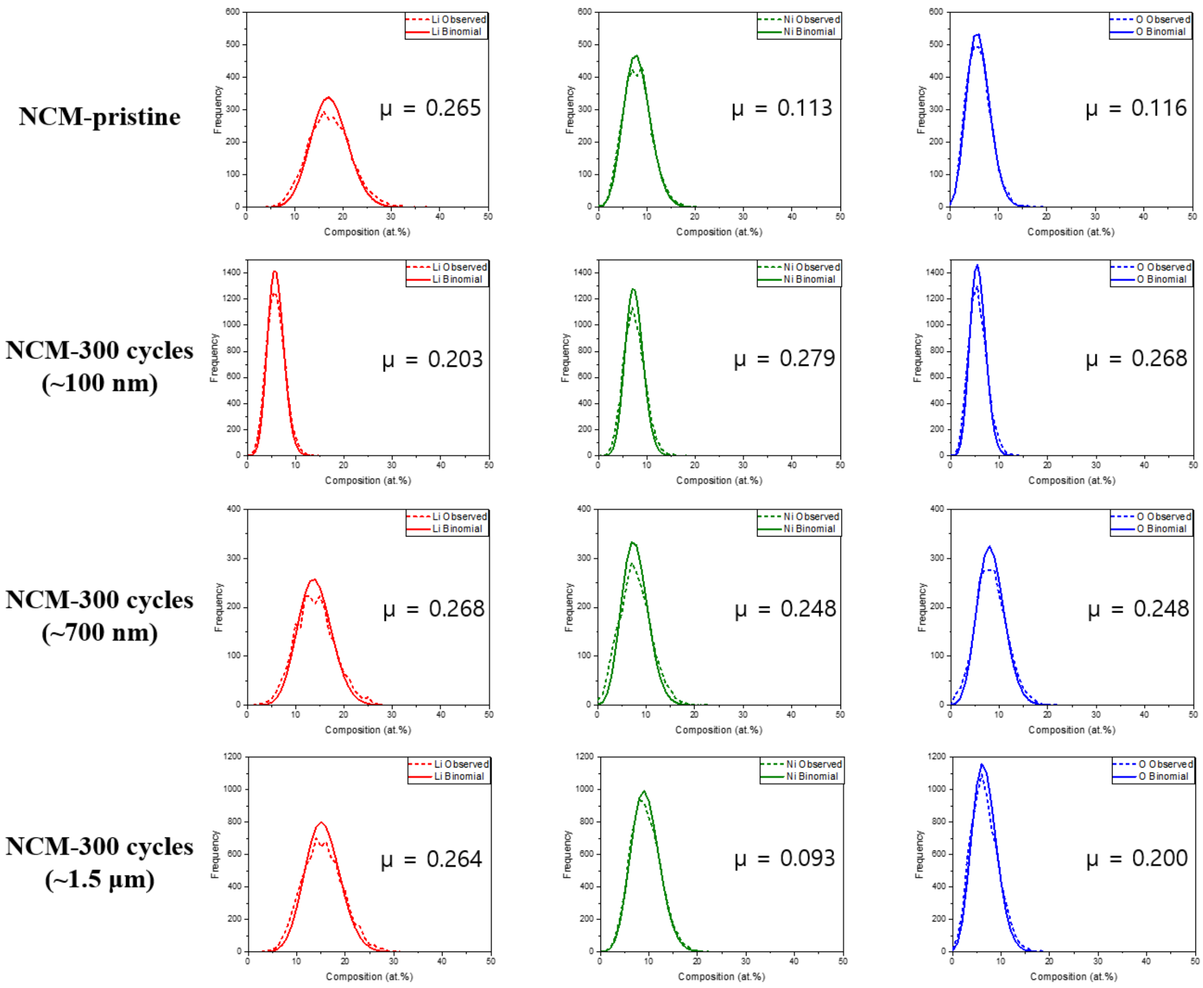

Supplementary Fig. 7. Compositional homogeneity according to a frequency distribution analysis. This analysis of NCM shows a rather uniform distribution of the constituent elements Li, Ni, and O along the depth from the particle surface. A Pearson coefficient (μ) far from 1.0 indicates a homogeneous distribution, and the observed frequency distribution is almost a binomial distribution.

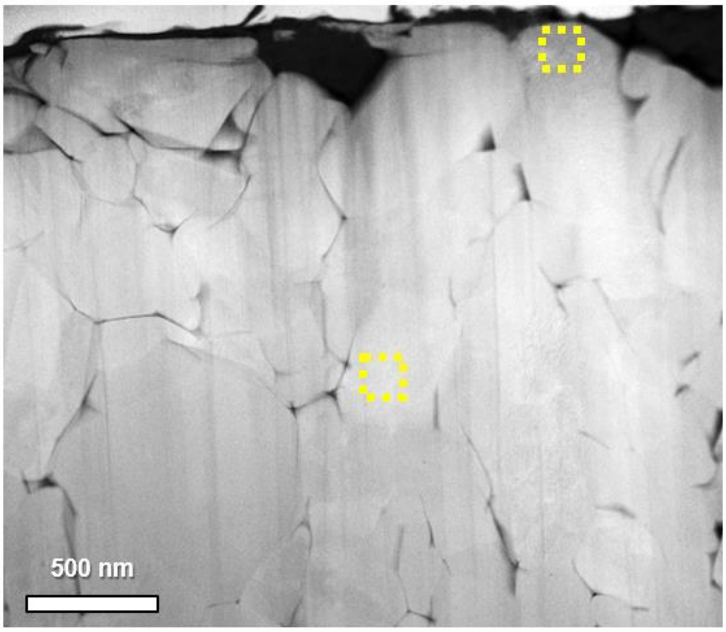

Supplementary Fig. 8. Low-magnification STEM-HAADF images of NCM-pristine. The regions displayed in **Fig. 2** are indicated by yellow dotted squares.

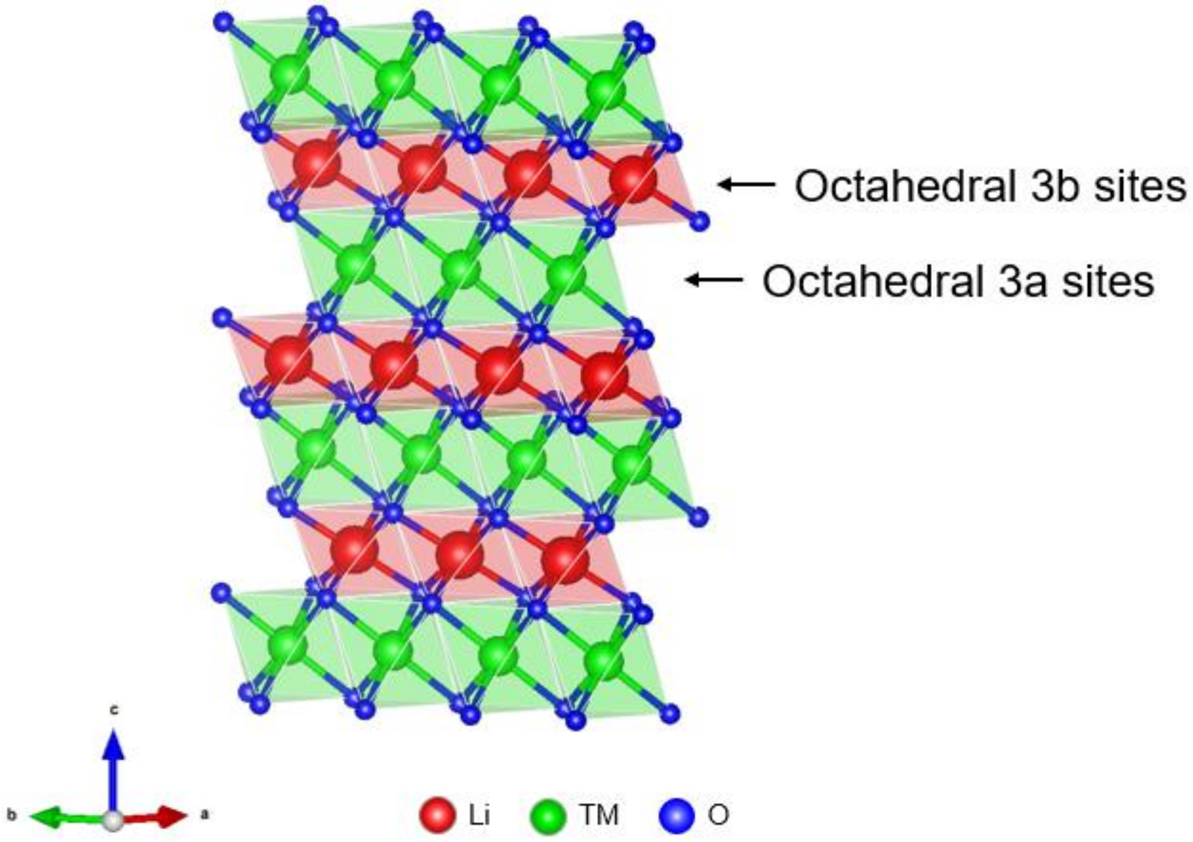

Supplementary Fig. 9. Schematic illustration of the positions of octahedral 3a and 3b sites.

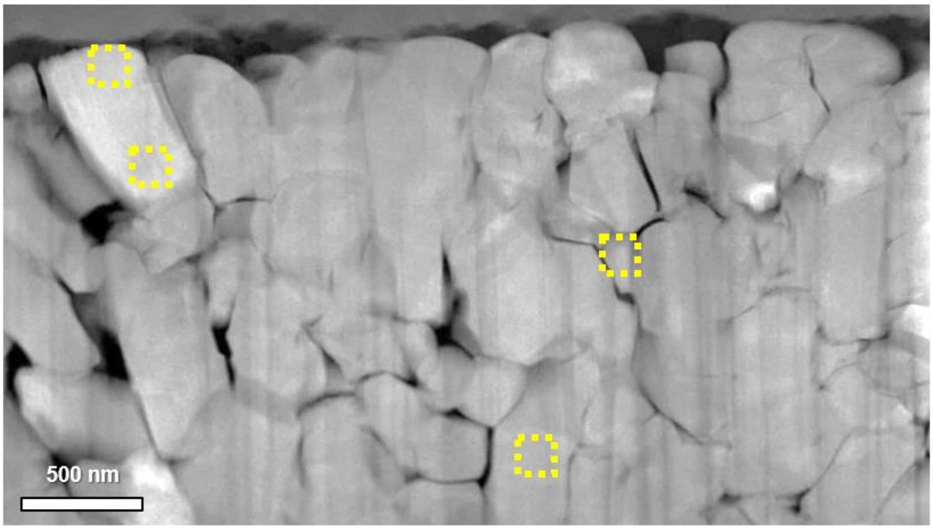

Supplementary Fig. 10. Low-magnification STEM-HAADF images of NCM-300 cycles, which shows crack formation or an increased gap between primary particles. The regions observed in **Fig. 3 and Fig. 4** are indicated by yellow dotted squares.

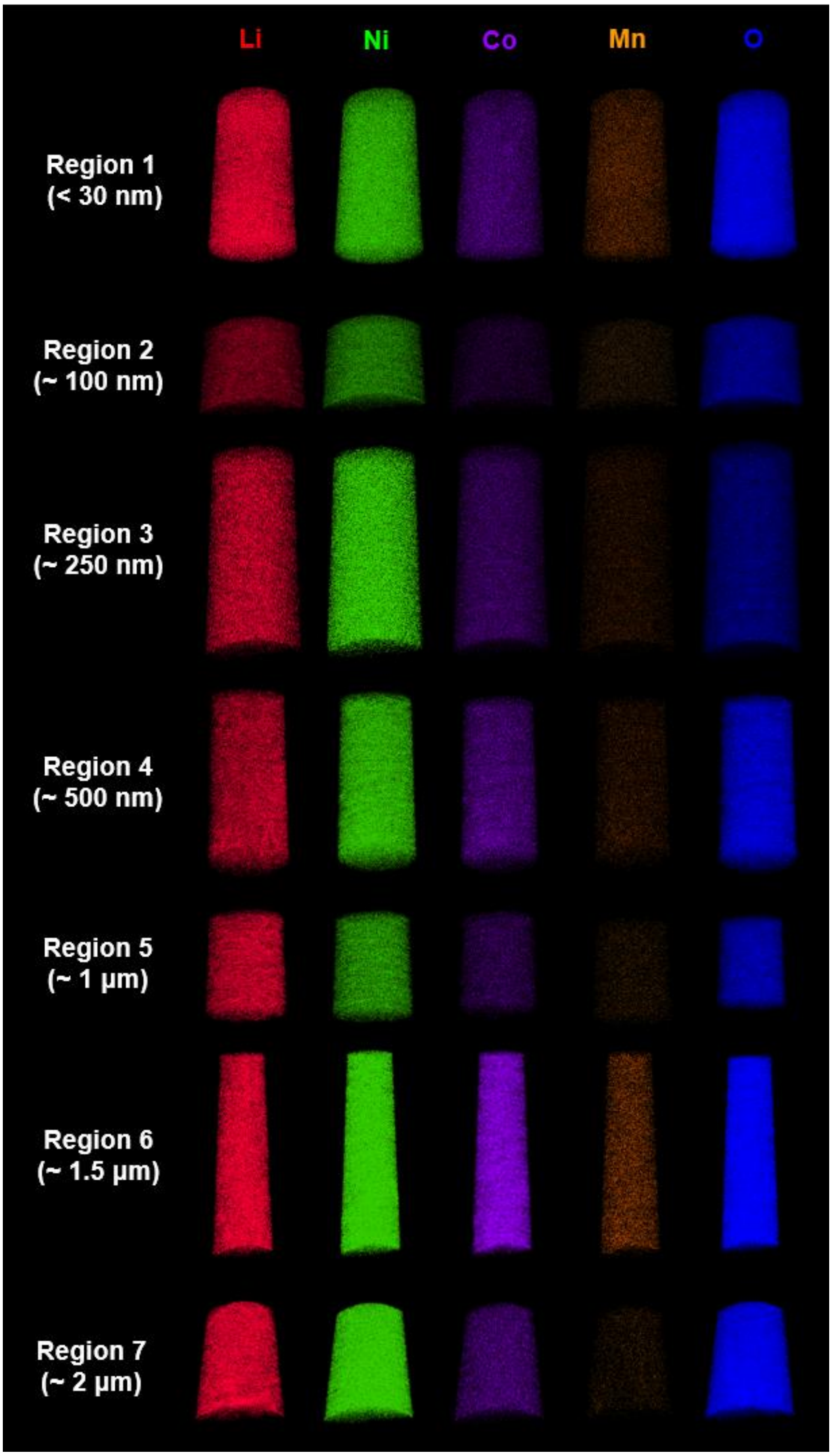

Supplementary Fig. 11. APT 3D atom maps of Li (red), Ni (green), Co (violet), Mn (orange), O (blue) along the depth of NCM-300 cycles, showing no elemental segregation.

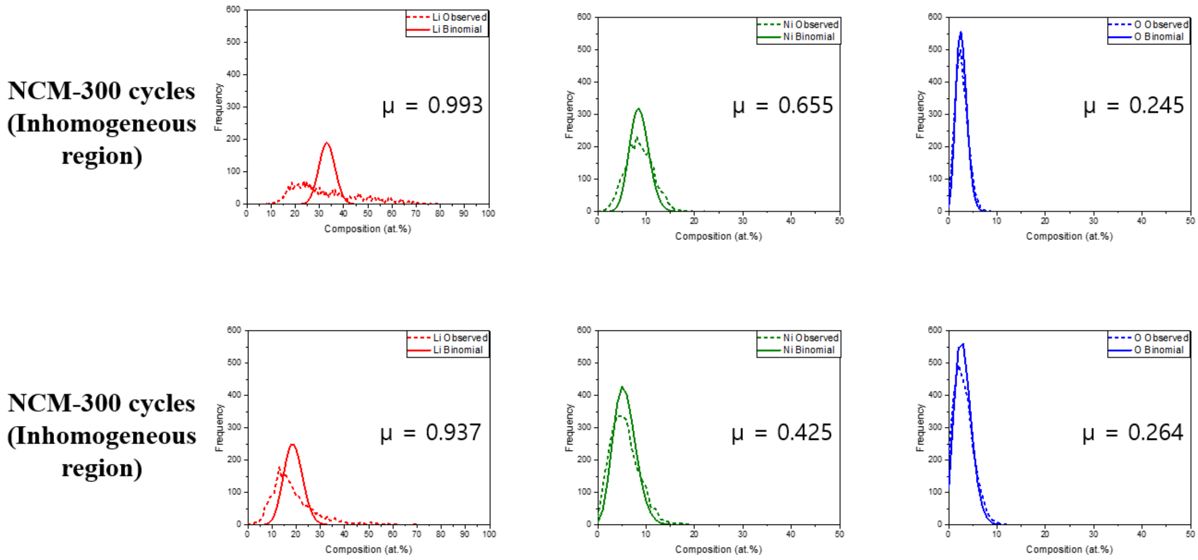

Supplementary Fig. 12. Compositional homogeneity according to a frequency distribution analysis of inhomogeneous regions. Frequency distribution analysis of the inhomogeneous region in NCM shows non-uniform distributions of the constituent elements. A Pearson coefficient (μ) close to 1.0 means a high degree of non-uniformity.

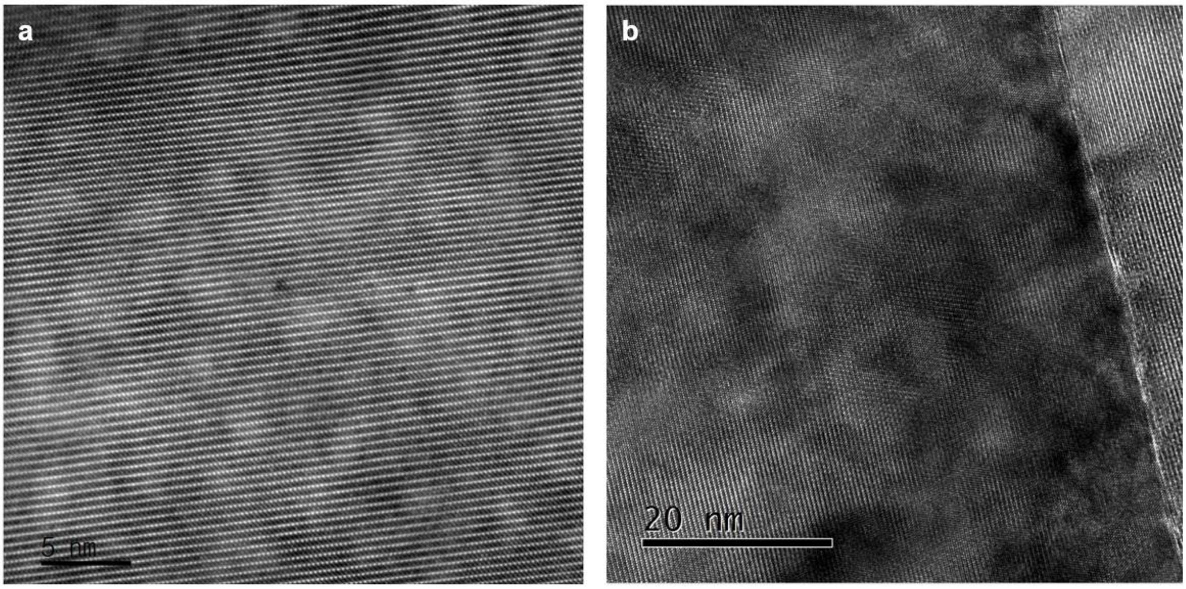

Supplementary Fig. 13. Examples of original STEM images before cropping. At low magnification, the structure is not clearly visible.

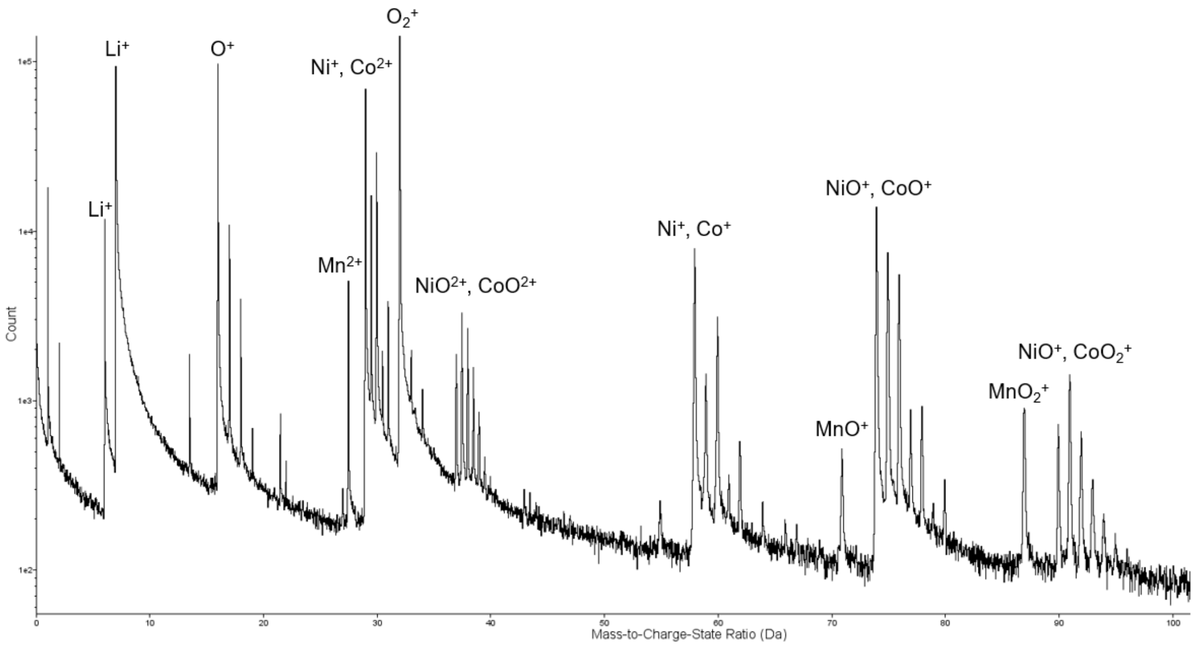

Supplementary Fig. 14. APT mass spectrum of NCM. The main peaks are labelled.

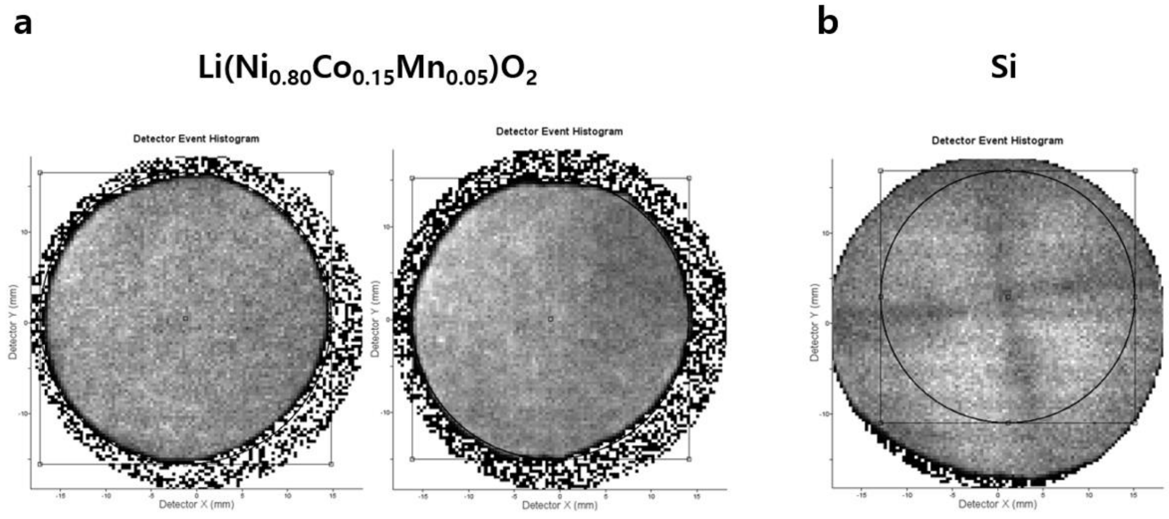

Supplementary Fig. 15. Detector event histogram of NCM and Si. Detector maps of NCM (a) and Si (b). A pole is not visible in the detector map of NCM, whereas a pole is clearly visible in that of Si.
